# Understanding Contact Electrification at Water/Polymer Interface

**DOI:** 10.34133/2022/9861463

**Published:** 2022-02-16

**Authors:** Yang Nan, Jiajia Shao, Morten Willatzen, Zhong Lin Wang

**Affiliations:** ^1^Beijing Institute of Nanoenergy and Nanosystems, Chinese Academy of Sciences, Beijing 101400, China; ^2^School of Nanoscience and Technology, University of Chinese Academy of Sciences, Beijing 100049, China; ^3^CUSTech Institute, Wenzhou, Zhejiang 325024, China; ^4^School of Materials Science and Engineering, Georgia Institute of Technology, Atlanta, Georgia 30332-0245, USA

## Abstract

Contact electrification (CE) involves a complex interplay of physical interactions in realistic material systems. For this reason, scientific consensus on the qualitative and quantitative importance of different physical mechanisms on CE remains a formidable task. The CE mechanism at a water/polymer interface is a crucial challenge owing to the poor understanding of charge transfer at the atomic level. First-principle density functional theory (DFT), used in the present work, proposes a new paradigm to address CE. Our results indicate that CE follows the same trend as the gap between the highest occupied and lowest unoccupied molecular orbitals (HOMO and LUMO) of polymers. Electron transfer occurs at the outmost atomic layer of the water/polymer interface and is closely linked to the functional groups and atom locations. When the polymer chains are parallel to the water layer, most electrons are transferred; conversely, if they are perpendicular to each other, the transfer of charges can be ignored. We demonstrate that a decrease in the interface distance between water and the polymer chains leads to CE in quantitative agreement with the electron cloud overlap model. We finally use DFT calculations to predict the properties of CE materials and their potential for triboelectric nanogenerator energy harvesting devices.

## 1. Introduction

Contact electrification (CE) is a well-known phenomenon describing how tribocharges are generated and distributed on contacting surfaces which is naturally present across all phases [1–3]. In spite of the long historical record, a detailed fundamental understanding of CE mechanisms, both extrinsic and intrinsic, remains indecisive [4–6]. As of today, two representative theoretical models have been established to obtain deeper insight into the charge transport: ion transfer and electron transfer between the contact interfaces [7–13]. Although absorbed ions (such as OH^−^) are important for surface-charge transfer, typical results indicate that they are not strictly necessary [14, 15]. An electron cloud model was proposed by Wang's group (also called a Wang transition) [3, 6, 16, 17] where the overlap of electron clouds between two atoms determines the strength of electron transfer between them. The electron transition model has a possible universal range of applicability yet its accuracy and reliability need further validation.

Since the complex interplay of interactions usually refers to a diverse range of effects in real materials, CE can normally be placed in three categories: CE of metal-metal, metal-insulator, and insulator-insulator interfaces [1–3, 6]. Previous studies suggest that CE for metal-polymer interfaces is susceptible not only to the values of the material energy gaps but also heavily affected by the interface distance, driving force, and contact stress at the contact region [18–21]; hence, it is still arduous to determine how electrons are transferred between polymers and other materials. Some research works argue that the electron acceptor orbital could be the lowest unoccupied molecular orbital (LUMO) on the polymer surface [19, 20]. However, we still have scarce knowledge of the CE mechanisms at water/polymer interfaces despite the fact that such phenomena are abundant in nature and technology. CE results in a complex behavior ranging from the microscopic level to the macroscopic level, and only advanced computational studies can provide an accurate understanding [22–24]. DFT is the most successful method to simulate ground-state properties of materials, which, meanwhile, provides a well description on molecular orbitals and energy of polymers [25–32]. We use DFT to determine the HOMO-LUMO gap, electron affinity, etc., so as to clarify the CE mechanism of water/polymer interfaces.

**Figure 1:**
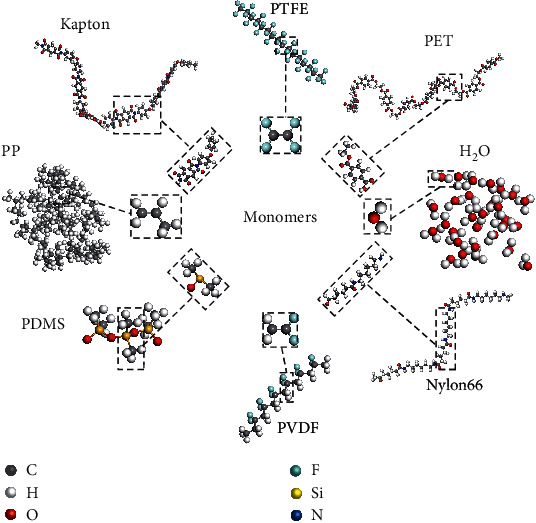
Sketch of seven polymers. Polytetrafluoroethylene (PTFE), polypropylene (PP), polyvinylidene difluoride (PVDF), polydimethylsiloxane (PDMS), Nylon 66, polyimide (Kapton), polyethylene terephthalate (PET), and water. The dashed areas depict their monomers.

In this work, some representative polymers containing different functional groups and repeat units are selected (Figure 1) to investigate CE in contact with water [3]. The dependence of charge transfer on water layer, length of the molecular chain, contact modes, and electrostatic potential, as well as WF before and after CE, are systematically discussed. Our findings indicate that when water comes in contact with different polymers, electron transfer occurs almost exclusively at the water/polymer interface, and only the outmost layer of water contributes. The surface HOMO-LUMO gap states are shown to be electron acceptors; in particular, a large HOMO-LUMO gap is highly beneficial for electron transfer. In this work, we aim to comprehensively discuss CE at water/polymer interfaces subject to ideal atomic order conditions leaving out ion concentration and geometry effects (such as contact angle), as we do not expect the latter assumptions to alter the general conclusions [1, 21].

## 2. Results

### 2.1. Effect of the Amount of Water and the Polymer Length on Charge Transfer

**Figure 2:**
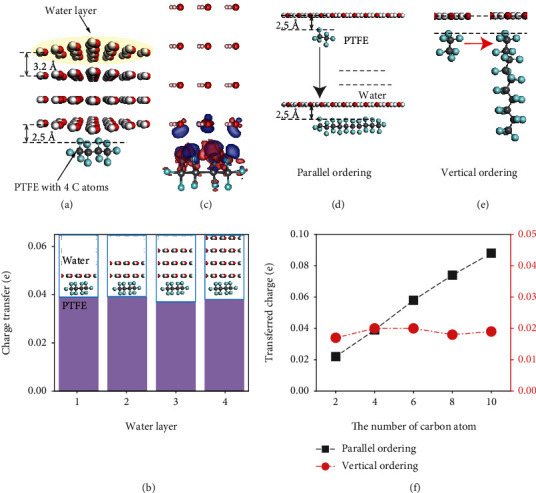
The effect of the number of water layers and the length of the chain on charge transfer. (a) The side view of a PTFE with four C atoms after CE with four layers of water. The distance between two water layers is 3.2 Å while the distance between a water layer and the PTFE is 2.5 Å. (b) Sketches of a PTFE chain with four C atoms in contact with different number of water layers and the corresponding transferred charges. (c) The CDD of CE between a PTFE chain with four C atoms and water with four layers. (d) Configurations of different length of PTFE in contact with one water layer in parallel and (e) vertical ordering. (f) The relationship between the charge transfer and different length of PTFE in contact with water at forms of parallel and vertical ordering, respectively.

We first explore the effect of water layer on charge transfer. The water thickness is varied (from one layer to four layers) while the polymer length, specified by the number of carbon atoms, is maintained constant (Figure 2). In addition, the distance between the polymer and water surfaces is fixed to rule out the distance factor for the charge transfer (Figure 2(a)). The distance between the water layer and PTFE is controlled by constraining the C atoms after full structure optimization. As shown in Figure 2(b), the difference in charge transfer with increased number of water layers for the same PTFE chain is ignorable. Moreover, charge density difference (CDD) is only observed at the outmost layer between water and PTFE (Figure 2(c)).

After that, two types, for the polymer chain parallel and vertical to the water surface, respectively, are analyzed to study the length effect of the PTFE chain on CE [33]. The length of a water layer, the number of water layers, and the distance between the water surface and the PTFE chain are all fixed. Figure 2(d) shows a PTFE chain, from two C atoms to ten C atoms' length, arranged parallel to the water layer. Similar to the case with CE for parallel alignment, Figure 2(e) displays the change of PTFE length for vertical positioning of the PTFE chain with respect to the water layers. Charge transfer for a water layer and a PTFE chain (both parallel and vertical ordering) for different lengths are depicted in Figure 2(f). Evidently, the charge transfer is proportional to the contact area.

### 2.2. Effect of Saturation on Charge Transfer

**Figure 3:**
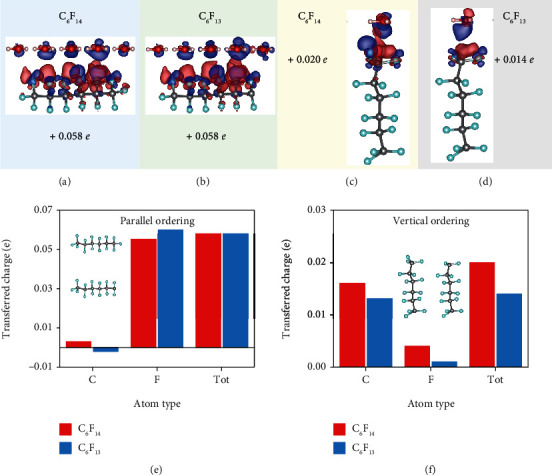
The effect of saturation on charge transfer. The charge transfer between PTFE and water in parallel ordering when the PTFE is (a) saturated and (b) unsaturated. The charge transfer between PTFE and water in vertical ordering when the PTFE is (c) saturated and (d) unsaturated. The corresponding total charge transfer for atoms of C and F (e) in parallel ordering and (f) vertical ordering, respectively.

We next consider the influence of saturation on CE, i.e., the difference in CE between saturated PTFE (that is, C_6_F_14_) and unsaturated PTFE (C_6_F_13_) in contact with water. Except for chains with different lengths, saturation in a polymer material is also diverse, which may have an influence on charge transfer. Here, for each aforementioned polymer/water type, we investigate the influence of saturation on charge transfer. The length of the PTFE chain, the amount of water, and the average distance between PTFE and water remain unchanged. For parallel ordering, charge transfer of saturated PTFE is equal to that of unsaturated PTFE (Figures 3(a) and 3(b)). However, the CDDs at the same location behave slightly different. Specifically, the CDD of water close to the end of unsaturated PTFE (C atom terminated side) lose more electrons than that of saturated PTFE. For vertical ordering, the amount of charge transfers of saturated PTFE CE (Figure 3(c)) is larger than that of unsaturated PTFE (Figure 3(d)). Meanwhile, the CDD of unsaturated PTFE (Figure 3(c)) shows a significant difference compared to the CDD of saturated PTFE (Figure 3(d)) where the C atom tends to obtain more electrons. For both saturated and unsaturated PTFE, CDDs are only found in the surface region between PTFE. Notably, for different PTFE chains of the same length, charge transfer for parallel ordering of water and PTFE layers shows larger values than for vertical ordering regardless of whether PTFE is saturated or unsaturated.

Our results reveal that charge transfer for parallel ordering of water and the polymer chain is the same whether the polymers are saturated or unsaturated. To demonstrate this, we calculate the charge transfer for each atom type in PTFE. As for parallel ordering (Figure 3(e)), the charge difference of F atoms is close to the total charge difference; thus, F atoms contribute the most to charge transfer. C atoms in saturated PTFE accept electrons while C atoms in unsaturated PTFE lose electrons. The absence of a F atom at the end of the PTFE chain creates a dangling bond on the C atom, whereby electrons accumulate more around this C atom than the C atom in the same position of saturated PTFE. As a result, the ability of the C atom to accept electrons is decreased. In addition, electrons on C atoms shift towards the nearby F atoms, leading to charge rearrangement in PTFE. This conclusion is also applicable to vertical ordering. The calculated amount of charge densities suggests that the electron density changes mostly near the C atoms for vertical ordering (Figure 3(f)). Because of the dangling bond in unsaturated PTFE, the C atom at the end cannot accept as many electrons as the C atom in saturated PTFE does, which causes a decrease in the total charge transfer for unsaturated PTFE in vertical ordering. We can summarize that the F atom is dominant for parallel ordering of water and PTFE while the C atom is dominant for vertical ordering, and the domination of F atoms induces more charge transfer. This provides evidence that the transferred charges increase with the increase of the PTFE length for parallel ordering of water and PTFE while charge transfer is independent of the PTFE length in for vertical ordering (Figure 2(f)). Our numerical calculations show a different mechanism compared to CE of polymer-metal interfaces where in the latter case, vertical ordering contributes more to CE than parallel ordering and the unsaturated chain is the main factor for CE owing to the dangling bond [33].

### 2.3. Electron Transfer Behaviors of Other Polymers

**Figure 4:**
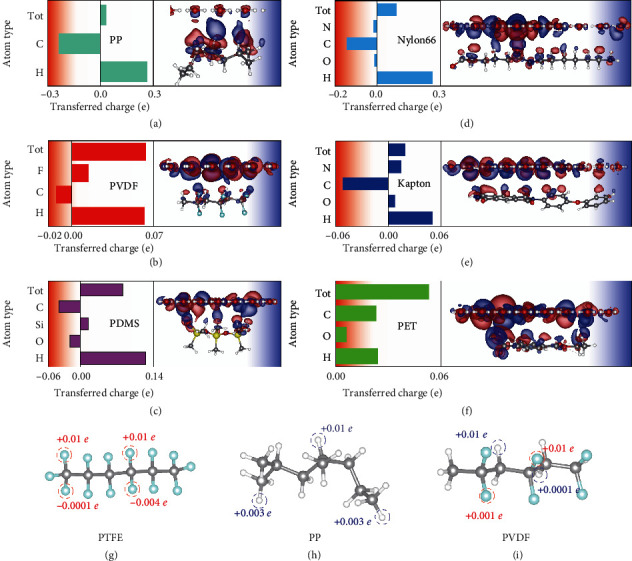
Charge transfer of different atoms and CDD for (a) PP, (b) PVDF, (c) PDMS, (d) Nylon 66, (e) Kapton, and (f) PET. Note that the pillar represents the total transferred charges for an atom in polymers. Charge differences of selected atoms (in the dashed circle) for (g) PTFE, (h) PP, and (i) PVDF after contact with water, respectively. And their contact configurations are shown in Figure 3(a) and (a, b), respectively.

We further calculate the charge transfer of each element and CDD for the other 6 polymers which are polypropylene (PP), polyvinylidene difluoride (PVDF), polydimethylsiloxane (PDMS), Nylon 66, polyimide (Kapton), and polyethylene terephthalate (PET), respectively (Figures 4(a)–4(f)). In addition, the optimized water layer remains constant so as to observe CDD most clearly and evaluate the charge difference of each atom in polymers before and after contacting with water. The distance between the water layer and the relaxed polymer is determined by the energy minimal state according to geometry optimization. For PP (Figure 4(a)), the electronic structure is rearranged after contact with water to reach a new state of charge stability. As a result, the charge differences of C atoms and H atoms are much larger than total charge transfer. Besides electrons transferred from water, some electrons in C atoms move to H atoms, leading to the increase of electrons of H atoms even some of the atoms are far from the water layer (Figure 4(h)). Differently, in the PTFE model, F atoms that contact water directly obtain electrons while lost electrons for F atoms on the other side (Figure 4(g)). However, F and H atoms in PVDF all obtain electrons (Figures 4(f) and 4(i)). The results illustrate charge transfer depends intensively on the bonding styles in polymers; the same element bonding with different atoms induces different charge distributions.

Electronic structure rearrangements are also found in PDMS, Nylon 66, and Kapton. At the same time, the charge distribution in water will also reach a new state according to the CDDs. In PDMS structure, O atoms lost electrons while their neighbors, Si and H atoms, obtain electrons (Figure 4(c) and Figure S1a) as a result of charge rearrangement after contact with water. As expected, the same elements in different polymers have different contributions. To be more specific, N atoms lost electrons in Nylon 66 (Figure 4(d) and Figure S1b) while obtaining electrons in Kapton (Figure 4(e) and Figure S1c). Inversely, O atoms obtain electrons in Nylon 66 but lost electrons in Kapton. Though all the elements obtain electrons in PET, the bonding effect on charge transfer is adaptable. The selected C atom bonding with H atom obtains more electrons than that of C bonding with the identical atom. We notice that the total transferred charge is the sum of charges obtained by each element; this is because of the planer structure of PET and the alignment of monomer comparing with other polymers. In addition, the O atom in PET closes to water shows a more complicated CDD than O that is relatively far from water, and the calculated charge differences denote the same element in different positions obtains different charges (Figure S1d), which could be a reasonable demonstration for the inhomogeneous charge distribution in actual applications. Besides, we confirm that the increased total electrons in polymers are totally obtained from water, which indicates the charge conservation in our system (Table S1). Our results reveal that the charge transfer of atoms in polymers during CE is dramatically influenced by bonding style, functional group, and position. The electronic structures and charge distributions of polymers will be influenced by the induced electrons.

### 2.4. Effect of Work Function and Interdistance

**Figure 5:**
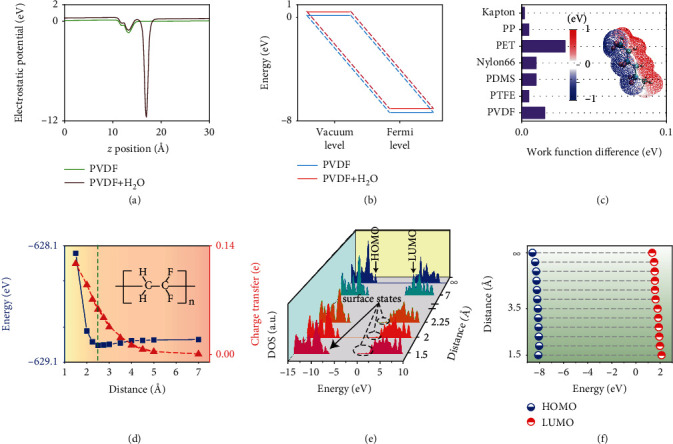
(a) The average electrostatic potential along the *z* axis before and after PVDF contacting with water. (b) The difference between the vacuum level and the Fermi level of PVDF after contacting with water. (c) The comparison of work function differences of polymers in this study after contacting with water. Inset shows the electrostatic potential mapping of PVDF. (d) The relationship between contacting distance and the system energy and the corresponding charge transfer of PVDF. (e) The DOS at the determined state of the change of contacting distance. (f) The variations in HOMO and LUMO levels as function of the contacting distance.

The average electrostatic potential of PVDF and PVDF with water along the *z*-direction is further calculated. The interface potential of PVDF increases slightly (at the location of 15 Å) after contacting with water (Figure 5(a)). In addition, the vacuum level difference comes from the change of the electrostatic potential in the vacuum area (0-10 Å and 20-30 Å). The changes of vacuum level and Fermi level show the charge transfer behavior after contacting with water, and the increase of Fermi level suggests the enlarged barrier for electrons (Figure 5(b)). The enlarged barrier suggests polymer with transferred electrons reached a stable state, hinders generated charge via CE flow back to the water, which agrees well with the experimental result that charge generated by CE retained [16]. In addition, we discuss the work function difference before and after CE. The work functions of polymers are all increased, and the increased work function means polymers play a role to accept electrons during CE, indicating the tendency that electrons transferred from water to the polymer and trapping near the surface of the polymer (Figure 5(c)). The inset displays the electrostatic potential mapping of PVDF; the positive potential of H atoms means they are more attractive to the electrons, which is consistent with the above results (Figure 4(b)). However, the work function difference cannot explain the discrepancy in the amount of charge transfer by different polymers, nor can it explain the relationship between charge transfer and the interface distance (Figure 5(d)). As is shown in Figure 5(d), the total energy of the system decreases at first because of the weakening of repulsive energy between atoms with the increase of interface distance to the equilibrium state (2.5 Å, the position at the green dashed line). As the interface distance increases continuously, the attractive effect is dominant and the system energy increases at first then tends to be stable. Nevertheless, the transferred charge decreases along with the increase of contact distance. The results can be well explained by the electron cloud overlap model [3, 16, 34]. Once the polymer and water are brought to contact with each other, their electrons clouds start to overlap, and electrons are hopping from water to the polymer to reach a steady electronic system. Decreasing with the interface distance, the overlapped part of electrons is enlarged, resulting in more charge transfer from water to the polymer. Other polymers also show the same tendency for the energy and charge transfer change with the contact distance (Figure S2a-c). These common phenomena are compatible with the experimental results that the current that water contacts the polymer is much higher than the current that water leaves the polymer [35], inspiring that increasing the effective pressure to shorten the distance between atoms is a promising strategy to enhance polymer-water CE.

To better understand the charge transfer behavior, we calculate DOS at different contact distances (Figure 5(e)). At the mostly closed contacting distance, surface states are observed near both HOMO and LUMO levels, suggesting electrons are transferred to the HOMO-LUMO gap especially near frontier molecular orbitals (FMOs). With the interface distance increases up to the equilibrium state, surface states are found only near LUMO, while it is not distinct near HOMO. Gradually, surface states vanish with the contact distance increases to infinite, which means polymer and water are brought to separate with each other. The change of HOMO and LUMO levels with contact distance is evaluated (Figure 5(f)); the levels of both HOMO and LUMO are increased because of inducing the electrons, which also proves that the electronic structure will be affected by the induced electrons, explaining for electronic configuration rearrangement and charge redistribution. Larger transferred electrons correspond to higher increases of HOMO and LUMO levels. Surface states are observed in PP and PDMS as well (Figure S2d-e); meanwhile, the HOMO and LUMO levels are increased by the transferred electrons (Figure S2g-h). Though surface state cannot be observed in Kapton at any contacting distance with water (Figure S2f), its HOMO and LUMO levels are greatly affected (Figure S2i). This is because, compared with other polymers, Kapton has a small HOMO-LUMO gap; the introduced electrons degenerate to HOMO and LUMO levels, leading to more increase of FMO levels. We can conclude that, for the polymer-water CE, electrons are transferred from water to the surface state in the HOMO-LUMO gap of polymer, and the electronic structures are influenced as well as the FMO levels are increased.

### 2.5. Determining Factor on Charge Transfer in Polymer/Water CE

**Figure 6:**
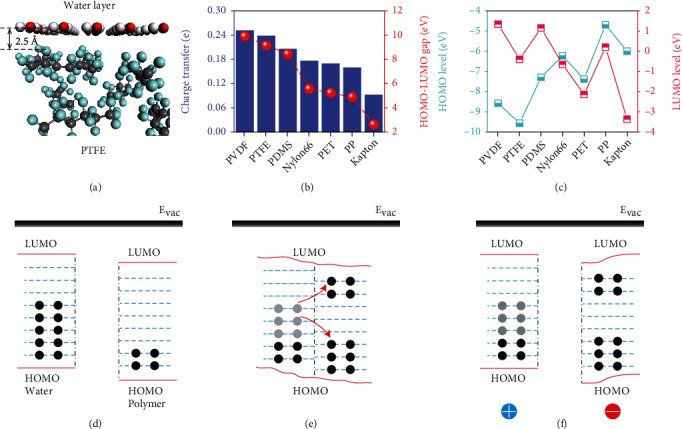
(a) The configuration of amorphous PTFE in contact with water layer, and the average distance between them is 2.5 Å. (b) The relationship between charge transfer and the HOMO-LUMO gap of amorphous polymers. (c) The HOMO and LUMO levels of each isolated polymer. The order of polymers is arranged according to their ability to accept electrons in (b). Schematics of level occupations of water and polymers (d) before contact, (e) in contact, and (f) after contact. Here, *E*_vac_ represents the vacuum energy level.

We then construct amorphous structures for each polymer (Figure S3) to evaluate the abilities that polymers obtain electrons from water; meanwhile, the amount of water keeps constant, and the atomic configurations of both top views and side views are displayed in Figure S3. The average distance between the water layer and the topmost atoms of the polymers is kept constant (Figure 6(a) and Figure S4). As shown in Figure 6(b), the order of obtaining electrons from the water has the same tendency as the HOMO-LUMO gap of polymer, illustrating a compact relationship between the capability of polymers to capture charges and the HOMO-LUMO gap. Notably, the order we calculated agrees well with the experimental result [36], proving the accuracy of our conclusion. Furthermore, the HOMO and LUMO levels of polymers are compared (Figure 6(c)). However, both HOMO and LUMO levels are irrelevant to the order that polymers obtain electrons shown in Figure 6(b). Indicating charge transfer in polymer-water CE depends strongly on the width of the HOMO-LUMO gap while independent of the HOMO and LUMO levels of polymer.

At last, we propose a theoretical model to reveal the underlying mechanisms of polymer-water CE (Figures 6(d)–6(f)). As polymers and water are separated from each other (Figure 6(d)), there is no charge transfer because the interface distance is too large, and the electron clouds of polymer and water have no overlapping areas. When the water is brought to contact with the polymer (Figure 6(e)), electron clouds on their surface start to overlap, leading to electrons hopping to the surface state of the polymer. Electrons are transferred to the HOMO-LUMO gap of polymers whereby they move towards the vicinity of HOMO and LUMO. After CE (Figure 6(f)), the induced electrons are retained at the surface states of the polymer by the enlarged barrier. Besides, the maintained electrons induce the distribution of electronic structure at the interface and the augment of HOMO and LUMO levels to reach a stable state. As a result, polymer is receiving electrons to become negative, while water is losing electrons to become positive. A wider HOMO-LUMO gap provides more surface states for accepting electrons from water, which explains the polymer order of the ability to obtain electrons as shown in Figure 6(b). Admittedly, the amount of transferred charge *Q* has the expression of *Q* = *σA*, where *A* represents the effective contacting area between polymer and water and *σ* is the surface charge density. Contacting areas of seven structures in the present study are constructed to be constant; thus, the charge transfer is dictated by *σ*. According to the expression of surface charge density [37], *σ* in this study can be derived as

(1) $$\begin{array}{c} \sigma ={eD}_{s}\left[\frac{E_{\text{gap}p}-E_{\text{gap}w}}{\left(1+e^{2}{dD}_{s}/\varepsilon_{s}\right)}\right], \end{array}$$

where *D*_*s*_, which can be detected in experimental, suggests the uniform distance of surface states per unit area per electronvolt; *ε*_*s*_ is the permittivity; *d* is the contact distance; *E*_gap*w*_ and *E*_gap*p*_ are HOMO-LUMO gaps of polymers and water, respectively. It is important to keep in mind that *E*_gap*w*_ in this study, which varies with the status of water, is constant. The charge transfer for a certain polymer is arguably increased corresponding to the decreasing of *d* according to Equation (1), which agrees exactly with simulation results (Figure 5(d)). As *d* is restricted, charge transfer is rigorously steered by HOMO-LUMO gap of the polymer (Figure 6(a)). Moreover, considering the area factor [21], charge transfer should be expressed as

(2) $$\begin{array}{c} Q={eD}_{s}\left[\frac{E_{\text{gap}p}-E_{\text{gap}w}}{\left(1+e^{2}{dD}_{s}/\varepsilon_{s}\right)}\right]\cdot \pi {\left(\frac{3V}{\pi \tan^{2}\theta /2\left(3/\sin\theta -\tan\theta /2\right)}\right)}^{2/3}, \end{array}$$

where *V* represents the volume of the water and *θ* is the contact angle.

## 3. Discussion

In this work, we have comprehensively studied the CE mechanism at water/polymer interfaces through density functional theory. Different from the CE mechanism of metal-metal and metal-insulator interfaces, the surface HUMO-LUMO gap state is confirmed to be the electron acceptor at the water/polymer interface. When water is in contact with a polymer, only electrons located on the surface (outmost layer) of water are transferred. In particular, if the polymer chains are parallel to the contact interface, the transferred electrons are directly proportional to the chain length reflecting the proportionality effect to the effective contact area. Electron transfer of saturated and unsaturated polymer is clearly distinguishable for vertical ordering while for parallel ordering, it is ignorable. On the other hand, we note that electron transfer is strongly associated with the functional groups and atom locations rather than the electronegativity of the atom species contained in the polymers.

Additionally, no single factor involved, such as work function difference, HOMO, and LUMO levels, uniquely determines the ability of polymers to accept electrons from water. Our results show that electron transfer can lead to rearrangement of the electronic structure and increased HOMO and LUMO levels governed by the surface HUMO-LUMO gap state. It should be noted that electron transfer is sensitively related to the interface distance between water and the outmost atomic layer of polymer that maintains the energy system in dynamic equilibrium. This result is in complete agreement with the electron cloud overlap model. A comprehensive study of the CE mechanism at the water/polymer interface is essential for a better physical understanding of electrification systems which eventually provides better utilization of CE materials for energy harvesting devices involving interdisciplinary research between physics, materials science, energy science, and engineering.

## 4. Materials and Methods

### 4.1. DFT Parameters

All calculations are performed using the Vienna ab initio simulation package (VASP) [38]. Generalized gradient approximation (GGA) with Perdew-Burke-Ernzerhof (PBE) [39] is chosen to describe the exchange-correlation energy. A 400 eV cut-off energy is set for electric wave functions, and the conjugate gradient algorithm is applied for structure optimization with a convergence threshold at 10^−5^ eV per atom for energy and 10^−2^ eV Å^−1^ per atom for force. Broyden-Fletcher-Goldfarb-Shanno (BFGS) algorithm [40] is used to help the structure reach the state of minimized energy. Additionally, DFT-D2 method of Grimme [41] is implemented to account for the dispersive forces to give a correct description of van der Waals (vdW) interactions. Bader charge analysis [42] is carried out to quantify the transferred charge before and after CE. Several preprocessing and postprocessing tools are applied to fulfill this work [43, 44].

### 4.2. Dealing with Water and Polymer Model

We use crystallized water in the simulations which previously was demonstrated to have similar coordination numbers and electronic structures with amorphous arranged water molecules [21]. As for the polymers with large repeating units, their monomers are used to study charge transfer since electron distributions in insulator monomers are localized [20]. To evaluate the relationship between distance and charge transfer, water and polymer are first relaxed. Then, the distance between water and monomer is changed, and the atoms in the main chain are fixed while those in the branch are relaxed to keep the distance constant when performing further relaxation. The amorphous structures of polymers are constructed according to their real density (Table S2). In the calculation for obtaining seven polymers' ability to accept electrons, the same amount of water is utilized and the average distance between the water layer and the amorphous polymer is kept constant.

## Data Availability

All data are available in the main text or supplementary materials. All other relevant source data are available from the corresponding authors upon reasonable request.

## Supplementary Materials

Supplementary Materials Figure S1: the charge difference of select atoms after contact with water. Figure S2: the relationship among contacting distance and the system energy, as well as the corresponding charge transfer. Figure S3: the top and side views of the amorphous structures of seven polymers. Figure S4: configurations of one layer water contact with amorphous polymers. Table S1: total charges of polymer and water before and after contact with each other. Table S2: density of polymers to construct the amorphous structures.

## References

[1] Lowell J., Rose-Innes A. C. (1980). Contact electrification. *Advances in Physics*.

[2] Shinbrot T., Ferdowsi B., Sundaresan S., Araujo N. A. M. (2018). Multiple timescale contact charging. *Physical Review Materials*.

[3] Wang Z. L., Wang A. C. (2019). On the origin of contact-electrification. *Materials Today*.

[4] Shaw P. E. (1926). The electrical charges from like solids. *Nature*.

[5] Lacks D. J., Shinbrot T. (2019). Long-standing and unresolved issues in triboelectric charging. *Nature Reviews Chemistry*.

[6] Wang Z. L. (2020). Triboelectric nanogenerator (TENG)-sparking an energy and sensor revolution. *Advanced Energy Materials*.

[7] Diaz A. F., Wollmann D., Dreblow D. (1991). Contact electrification: ion transfer to metals and polymers. *Chemistry of Materials*.

[8] McCarty L. S., Winkleman A., Whitesides G. M. (2007). Electrostatic self-assembly of polystyrene microspheres by using chemically directed contact electrification. *Angewandte Chemie, International Edition*.

[9] McCarty L. S., Winkleman A., Whitesides G. M. (2007). Ionic electrets: electrostatic charging of surfaces by transferring mobile ions upon contact. *Journal of the American Chemical Society*.

[10] Kwetkus B. A., Sattler K. (1991). Contact charging of oxidized metal powders. *Zeitschrift fur Physik B: Condensed Matter*.

[11] Zhang Y., Shao T. (2013). Effect of contact deformation on contact electrification: a first-principles calculation. *Journal of Physics D: Applied Physics*.

[12] Abdelaziz K. M., Chen J., Hieber T. J., Leseman Z. C. (2018). Atomistic field theory for contact electrification of dielectrics. *Journal of Electrostatics*.

[13] Peng J., Kang S. D., Snyder G. J. (2017). Optimization principles and the figure of merit for triboelectric generators. *Science Advances*.

[14] McCarty L. S., Whitesides G. M. (2008). Electrostatic charging due to separation of ions at interfaces: contact electrification of ionic electrets. *Angewandte Chemie, International Edition*.

[15] Baytekin H. T., Baytekin B., Soh S., Grzybowski B. A. (2011). Is water necessary for contact electrification?. *Angewandte Chemie, International Edition*.

[16] Xu C., Zi Y., Wang A. C. (2018). On the electron-transfer mechanism in the contact-electrification effect. *Advanced Materials*.

[17] Lin S., Xu L., Xu C. (2019). Electron transfer in nanoscale contact electrification: effect of temperature in the metal-dielectric case. *Advanced Materials*.

[18] Kahn A. (2016). Fermi level, work function and vacuum level. *Materials Horizons*.

[19] Wu J., Wang X., Li H., Wang F., Yang W., Hu Y. (2018). Insights into the mechanism of metal-polymer contact electrification for triboelectric nanogenerator via first-principles investigations. *Nano Energy*.

[20] Wu J., Wang X., Li H., Wang F., Hu Y. (2019). First-principles investigations on the contact electrification mechanism between metal and amorphous polymers for triboelectric nanogenerators. *Nano Energy*.

[21] Sun M., Lu Q., Wang Z. L., Huang B. (2021). Understanding contact electrification at liquid-solid interfaces from surface electronic structure. *Nature Communications*.

[22] Louie S. G., Chan Y. H., da Jornada F. H., Li Z., Qiu D. Y. (2021). Discovering and understanding materials through computation. *Nature Materials*.

[23] Marzari N., Ferretti A., Wolverton C. (2021). Electronic-structure methods for materials design. *Nature Materials*.

[24] Fish J., Wagner G. J., Keten S. (2021). Mesoscopic and multiscale modelling in materials. *Nature Materials*.

[25] Hohenberg P., Kohn W. (1964). Inhomogeneous Electron Gas. *Physical Review*.

[26] Parr R. G., Yang W. (1989). *Density-functional theory of atoms and molecules*.

[27] Perdew J. P., Levy M. (1983). Physical content of the exact Kohn-Sham orbital energies: band gaps and derivative discontinuities. *Physical Review Letters*.

[28] Kohn W., Sham L. J. (1965). Self-consistent equations including exchange and correlation effects. *Physical Review*.

[29] Issa Y. M., Abdel-Latif S. A., El-Ansary A. L., Hassib H. B. (2021). The synthesis, spectroscopic characterization, DFT/TD-DFT/PCM calculations of the molecular structure and NBO of the novel charge-transfer complexes of pyrazine Schiff base derivatives with aromatic nitro compounds. *New Journal of Chemistry*.

[30] Abdel-Latif S. A., Moustafa H. (2017). Synthesis, characterization, electronic structure, and non-linear optical properties (NLO) of Mn(II), Co(II), Ni(II), Cu(II) and Zn(II) complexes with 5-phenylazo-8-hydroxyquinoline using DFT approach. *Applied Organometallic Chemistry*.

[31] Abdel-Latif S. A., Mohamed A. A. (2017). Synthesis, structure, spectroscopic properties and DFT studies on some 7-hydroxy-4-methyl-8-(arylazo)-2H-1-benzopyran-2-one and their complexes with some divalent transition metal ions. *Journal of Molecular Structure*.

[32] Darweesh A. F., Abd El-Fatah N. A., Abdel-Latif S. A., Abdelhamid I. A., Elwahy A. H. M., Salem M. E. (2021). Synthesis and DFT studies of novel aminoimidazodipyridines using 2-(3H-imidazo [4, 5-b] pyrid-2-yl) acetonitrile as an efficient key precursor. *Arkivoc*.

[33] Yoshida M., Ii N., Shimosaka A., Shirakawa Y., Hidaka J. (2006). Experimental and theoretical approaches to charging behavior of polymer particles. *Chemical Engineering Science*.

[34] Willatzen M., Lin Wang Z. (2018). Theory of contact electrification: optical transitions in two-level systems. *Nano Energy*.

[35] Zhu G., Su Y., Bai P. (2014). Harvesting water wave energy by asymmetric screening of electrostatic charges on a nanostructured hydrophobic thin-film surface. *ACS Nano*.

[36] Xu W., Zheng H., Liu Y. (2020). A droplet-based electricity generator with high instantaneous power density. *Nature*.

[37] Cowley A. M., Sze S. M. (1965). Surface states and barrier height of metal-semiconductor systems. *Journal of Applied Physics*.

[38] Kresse G., Furthmüller J. (1996). Efficient iterative schemes forab initiototal-energy calculations using a plane-wave basis set. *Physical Review B*.

[39] Perdew J. P., Burke K., Ernzerhof M. (1996). Generalized gradient approximation made simple. *Physical Review Letters*.

[40] Head J. D., Zerner M. C. (1985). A Broyden-Fletcher-Goldfarb-Shanno optimization procedure for molecular geometries. *Chemical Physics Letters*.

[41] Grimme S. (2006). Semiempirical GGA-type density functional constructed with a long-range dispersion correction. *Journal of Computational Chemistry*.

[42] Tang W., Sanville E., Henkelman G. (2009). A grid-based Bader analysis algorithm without lattice bias. *Journal of Physics: Condensed Matter*.

[43] Wang V., Xu N., Liu J. C., Tang G., Geng W. T. (2021). VASPKIT: a user-friendly interface facilitating high-throughput computing and analysis using VASP code. *Computer Physics Communications*.

[44] Lu T., Chen F. (2012). Multiwfn: a multifunctional wavefunction analyzer. *Journal of Computational Chemistry*.

