# Supplementary material for: Understanding Contact Electrification at Water/Polymer Interface
Source: Research (Wash D C). 2022 Feb 16;2022:9861463. doi: 10.34133/2022/9861463 (PMC8873953; doi:10.34133/2022/9861463)
Supplement: Supplementary Materials — Figure S1: the charge difference of select atoms after contact with water. Figure S2: the relationship among contacting distance and the system energy, as well as the corresponding charge transfer. Figure S3: the top and side views of the amorphous structures of seven polymers. Figure S4: configurations of one layer water contact with amorphous polymers. Table S1: total charges of polymer and water before and after contact with each other. Table S2: density of polymers to construct the amorphous structures. [file 9861463.f1.doc]

**Supporting Information File**

Understanding contact electrification at water/polymer interface

Yang Nan, Jiajia Shao, Morten Willatzen*, and Zhong Lin Wang*

*Corresponding author. Email: mortenwillatzen@binn.cas.cn, zlwang@binn.cas.cn


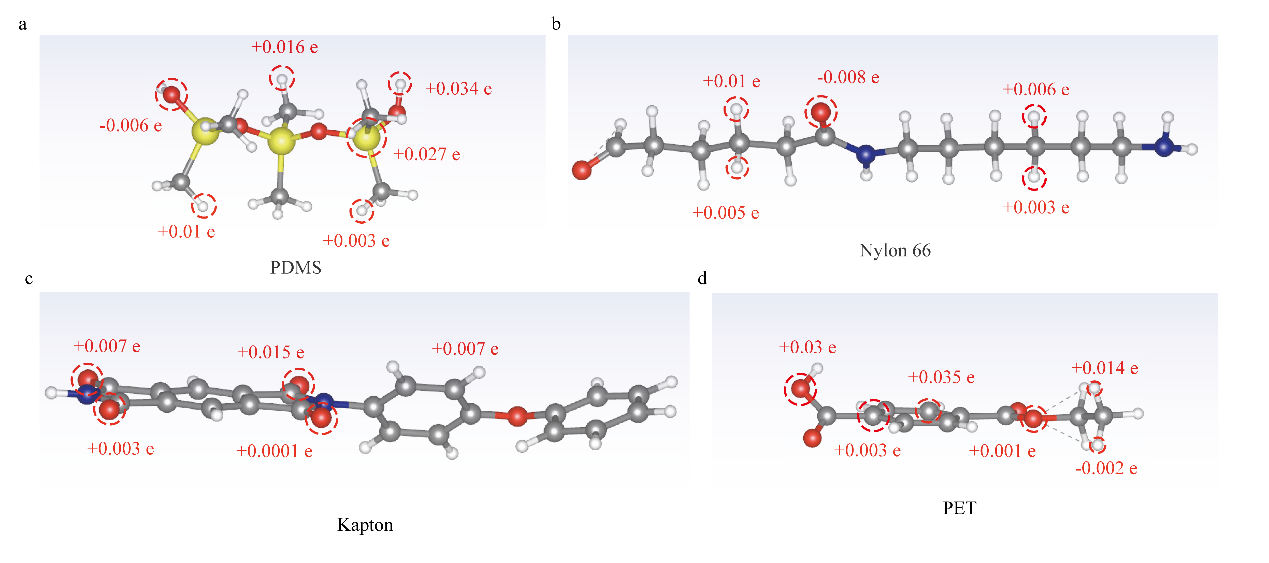


**Figure S1.** The charge difference of select atoms after contact with water for (a) PDMS, (b) Nylon 66, (c) Kapton and (d) PET respectively.


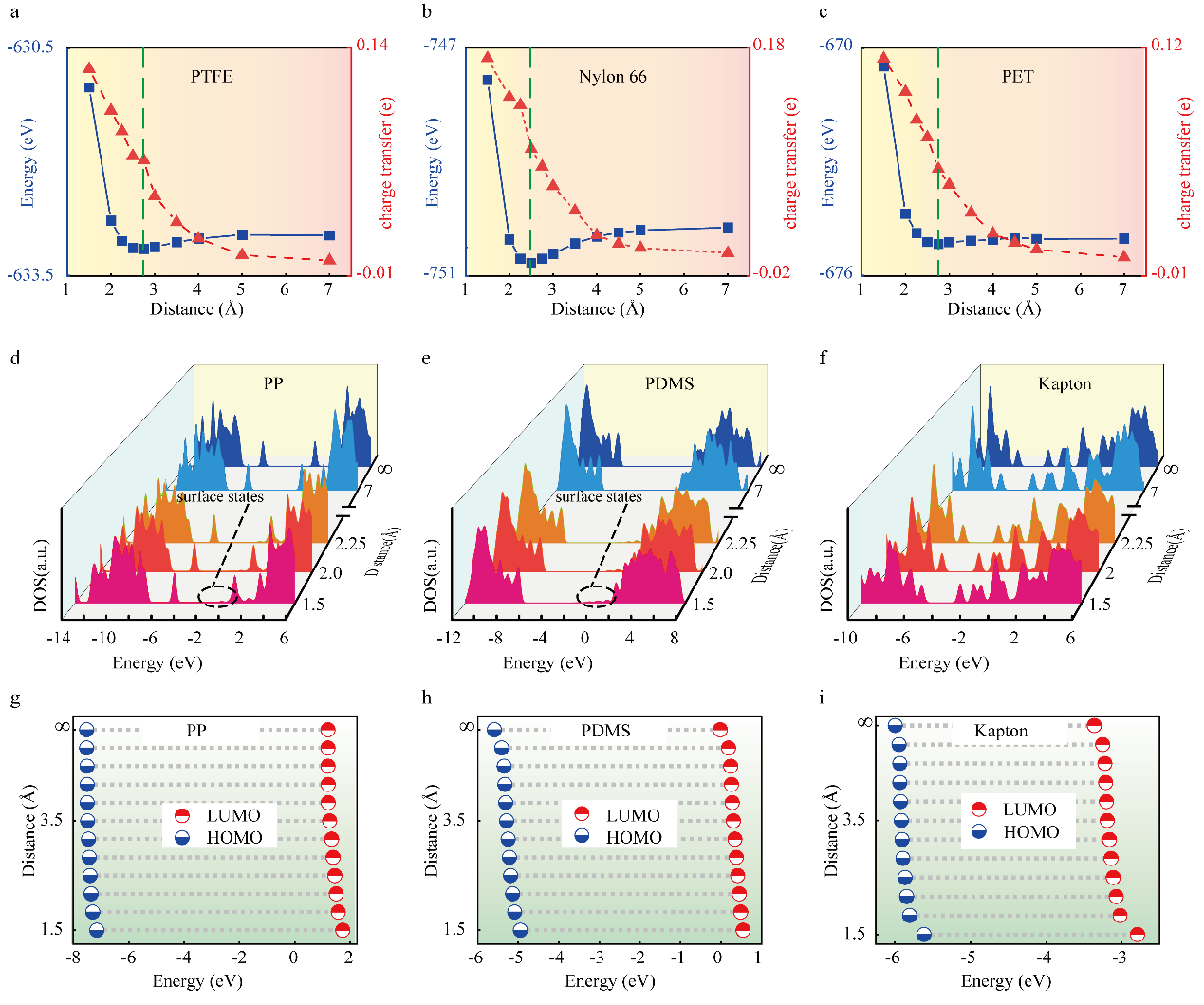


**Figure S2.** The relationship between contacting distance and the system energy and the corresponding charge transfer of (a) PTFE, (b) Nylon 66 and (c) PET. The DOS at the determined state of the change of contacting distance for (d) PP, (e) PDMS and (f) Kapton. The varieties of HOMO and LUMO level accomplish with the change of contacting distance for (g) PP, (h) PDMS and (i) Kapton.


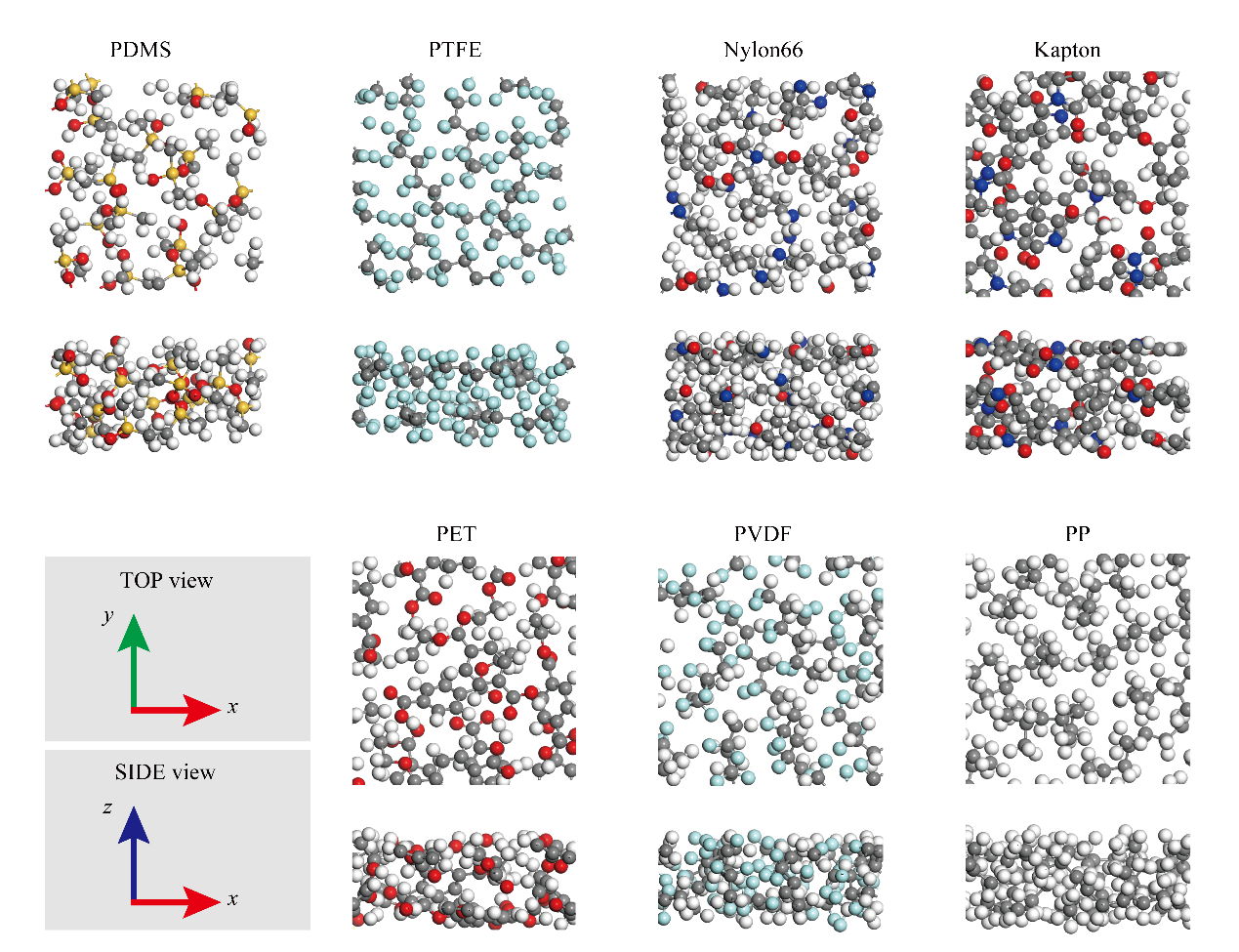


**Figure S3.** The top and side views of the amorphous structures of seven polymers which are PDMS, PTFE, Nylon66, Kapton, PET, PVDF, PP respectively.


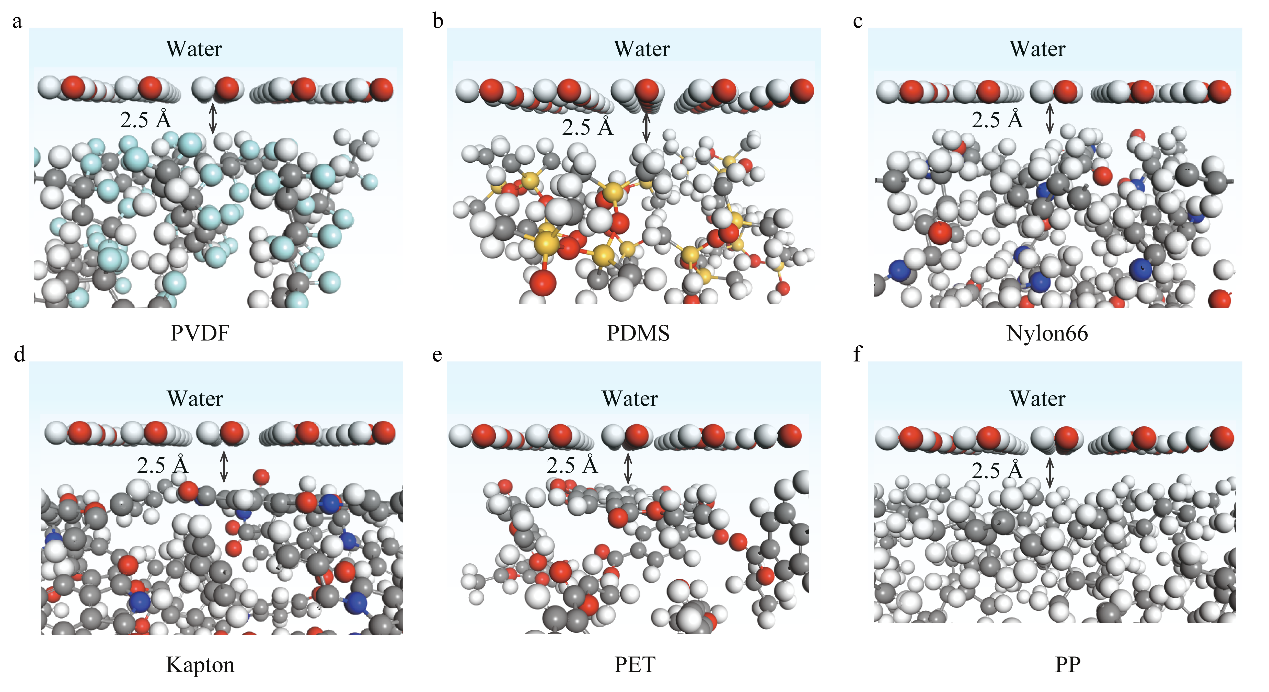


**Figure S4.** The configurations of one layer water contact with amorphous polymers which are PVDF, PDMS, Nylon66, Kapton, PET and PP, respectively. The average between water layer and topmost polymers keep constant.

**Table S1. The charge of polymer and water before and after contact.**

| **Name** | **Total charge before contact (e)** | | | **Total charge after contact (e)** | | |
| --- | --- | --- | --- | --- | --- | --- |
|  | **monomer** | **water** | **total** | **monomer** | **water** | **total** |
| PTFE | 122 | 288 | 410 | 122.06 | 287.94 | 410 |
| PDMS | 80 | 288 | 368 | 80.08 | 287.92 | 368 |
| Kapton | 140 | 288 | 428 | 140.02 | 287.98 | 428 |
| PET | 74 | 288 | 362 | 74.05 | 287.95 | 362 |
| PVDF | 74 | 288 | 362 | 74.06 | 287.94 | 362 |
| Nylon 66 | 94 | 288 | 382 | 94.09 | 287.91 | 382 |
| PP | 56 | 288 | 344 | 56.03 | 287.97 | 344 |

**Table S2. The real density of polymer to construct the amorphous structure.**

| **Name** | **Density (g/cm^3^)** |
| --- | --- |
| PTFE | 2.2 |
| PDMS | 1 |
| Kapton | 1.42 |
| PET | 1.38 |
| PVDF | 1.78 |
| Nylon 66 | 1.14 |
| PP | 0.90 |
